# Common Genetic Denominators for Ca^++^-Based Skeleton in Metazoa: Role of Osteoclast-Stimulating Factor and of Carbonic Anhydrase in a Calcareous Sponge

**DOI:** 10.1371/journal.pone.0034617

**Published:** 2012-04-10

**Authors:** Werner E. G. Müller, Xiaohong Wang, Vlad A. Grebenjuk, Michael Korzhev, Matthias Wiens, Ute Schloßmacher, Heinz C. Schröder

**Affiliations:** 1 ERC Advanced Investigator Grant Research Group at Institute for Physiological Chemistry, University Medical Center of the Johannes Gutenberg University Mainz, Mainz, Germany; 2 National Research Center for Geoanalysis, Chinese Academy of Geological Sciences, CHN-Beijing, China; University of Iowa, United States of America

## Abstract

Calcium-based matrices serve predominantly as inorganic, hard skeletal systems in Metazoa from calcareous sponges [phylum Porifera; class Calcarea] to proto- and deuterostomian multicellular animals. The calcareous sponges form their skeletal elements, the spicules, from amorphous calcium carbonate (ACC). Treatment of spicules from *Sycon raphanus* with sodium hypochlorite (NaOCl) results in the disintegration of the ACC in those skeletal elements. Until now a distinct protein/enzyme involved in ACC metabolism could not been identified in those animals. We applied the technique of phage display combinatorial libraries to identify oligopeptides that bind to NaOCl-treated spicules: those oligopeptides allowed us to detect proteins that bind to those spicules. Two molecules have been identified, the (putative) enzyme carbonic anhydrase and the (putative) osteoclast-stimulating factor (OSTF), that are involved in the catabolism of ACC. The complete cDNAs were isolated and the recombinant proteins were prepared to raise antibodies. In turn, immunofluorescence staining of tissue slices and qPCR analyses have been performed. The data show that sponges, cultivated under standard condition (10 mM CaCl_2_) show low levels of transcripts/proteins for carbonic anhydrase or OSTF, compared to those animals that had been cultivated under Ca^2+^-depletion condition (1 mM CaCl_2_). Our data identify with the carbonic anhydrase and the OSTF the first two molecules which remain conserved in cells, potentially involved in Ca-based skeletal dissolution, from sponges (sclerocytes) to human (osteoclast).

## Introduction

During the transition from the premetazoan to the metazoan multicellular organisms the toolkit for cell-cell and cell-matrix adhesion had to evolve allowing a coordinated and tuned interaction of cells into complex tissue units [1]. The sponges [phylum Porifera] had been, since the cell interaction studies of Wilson [2], a model system for investigations on morphogenetic processes in Metazoa. Later, the process of reaggregation of single cells to reconstitute functional systems tissue units had been studied in details by Moscona [3]. The first successful identification of purified proteins/molecules underlying the cell adhesion process in sponges had been achieved by Müller and Zahn [4] and Turner and Burger [5]; reviewed in Kuhns, et al. [6] and Müller [7]. Subsequently, the intracellular signal transduction pathways in sponges had been identified [1] allowing to place them to the genuine kingdom of Metazoa and, by that, establishing the monophyletic origin of all multicellular taxa [8]. By application of molecular clock calculations, based on protein-coding genes, the origin of Metazoa with the Porifera as the first taxon evolving for the hypothetical urmetazoan, had been calculated back to 650–665 million years [Myr] ago [9], a figure that had been confirmed by fossil records [10] with 635 Myr ago. These findings demonstrate that the first animals, the sponges, branched off from the Urmetazoa prior to the Marinoan glaciation (635 Myr ago), a period of worldwide glaciations “Snowball Earth” [11], during which the ocean had been silicon-rich [11]. In such an environment the two classes of siliceous sponges, the Hexactinellida and the Demospongia, evolved [12], while the class of Calcarea emerged later in a calcium-rich ocean; this shift in the composition of the ocean from silicon-rich to calcium-rich was the consequence of chemical weathering of calcium-silicate rocks.

The phylogenetic oldest classes of sponges, the Hexactinellida and the Demospongia, comprise an inorganic skeleton [spicule system], formed of amorphous silica, while the Calcarea stabilize their body with amorphous calcium carbonate [ACC] [13]. The formation of the siliceous spicules is reasonably well understood on the morphological, cell biological and molecular biological level. The siliceous spicules have either a monaxonal or a triaxonal shape (hexactinellids) or, as in demosponges, a monaxonal or tetraxonal architecture [14]. The formation of siliceous spicules, exemplarily studied at the model system *Suberites domuncula* (Demospongia), starts intracellularly in special cells, the sclerocytes, and is completed extracellularly [15]. The export of the immature spicules into the extracellular space occurs via an evagination process [16]. The inorganic silica polymer, also termed biosilica, is formed enzymatically via silicatein, an enzyme that belongs to the papain-like protease family [17]–[19] and follows the usual Michaelis-Menten kinetics [20]. In association with silintaphin-1 and silintaphin-2, silicatein represent the key structure-given proteinaceous scaffold around which biosilica is deposited [21]. The sponge biosilica is a hybrid material, formed from silica and proteinaceous material, very likely with silicatein as the major component.

In contrast to the siliceous spicules in demosponges, the formation of the calcareous skeletal elements in Calcarea is only understood on the chemical/physical-chemical level [22]. Initial observations [13] revealed that each single actine/ray of a calcareous spicule is produced by a few (around two) skeletal cells, the sclerocytes. Evidence has been presented, suggesting that the spatial arrangement of the sclerocytes determine the morphology of the spicules [23], [24]. Physicochemically, they are considered as a single calcareous crystal and their different rays start from a single organizing center. An important step towards an understanding of the crystallographic orientation of the calcite nucleus came from the studies of Addadi, et al. [25] and Heywood and Mann [26] who proposed that an organic polyanionic matrix forms the initial calcite nucleus from where the entire spicule is formed along a crystal lattice of the existing primordial spicule calcite [27]. The central part of the spicules is composed of a thin calcite that is embedded in a large layer of ACC which is surrounded by a thin calcite envelope [22]. The spicule is finally sealed by an organic sheath that is tightly associated with the predominantly inorganic central calcareous crystal(s) [24]. The nature of the proposed organic matrix in calcareous sponge spicules remained obscure. Already Haeckel [28] proposed that the inorganic crystallization process is driven by an organic component a “Central-faden”, a view that had been elaborated further by Weinschenk [29]. The latter author concluded that the organic matrix of the spicules decisively control their morphology and surface structures. Finally Aizenberg, et al. [30] demonstrated that the biogenic phase within those sponge spicules is rich in glutamic acid and hydroxyamino acids. Until now the nature of the organic material remained undetermined. Hence it remained unclear if the deposition of ACC is a process driven or controlled by an enzyme. It is discussed over 30 years that the enzyme carbonic anhydrase is somehow involved in the calcification of skeletal structures in Metazoa, e.g. crabs [31] or calcareous sponges [32]–[34].

In order to identify a protein interacting with an inorganic phase we have employed here for the first time the technique of phage display combinatorial libraries, which is based on the expression of random peptides that have the properties to bind to matrices, among them also to inorganic particles [35]. The sponge specimens used for the analysis were exposed to low concentrations of Ca^2+^ ions in order to assess the effect of the deprivation of this cation on the Ca-carbonate catabolism of the spicules. In nature – and this applied for sponge spicules also – anabolic processes, occurring during synthesis of a given biomineral, are intimately connected with catabolic processes. Applying this technique of phage display together with sponge cDNA libraries (EST [Expressed Sequence Tag] database) we identified two polypeptides that bind strongly to cleaned calcareous spicules from *Sycon raphanus*; the carbonic anhydrase and the osteoclast-stimulating factor [OSTF]. Since the enzyme carbonic anhydrase is reversibly catalyzing the interconversion of carbon dioxide and water to bicarbonate and protons, this process had been proposed to be involved in the cycling of calcareous sponge spicules [32]. Second, the OSTF is well known to be crucially involved in the development of the osteoclast cells which are controlling the dissolution of calcium hydroxyapatite [HA] in bone of vertebrates [36]. In the present study we propose that these two polypeptides are intimately involved in calcareous spicule metabolism, more specific in the dissolution of spicules. This conclusion is based on immunohistological localization studies performed with antibodies, raised against these two proteins.

## Materials and Methods

### Sponge

Specimens of *Sycon raphanus* (Porifera, Calcarea, Leucosolenida, Sycettidae) were collected in the Northern Adriatic Sea (Limski Chanel near Rovinj; Croatia) at depths between 2 to 7 m, growing as epibionts onto the mussel *Mytilus galloprovincialis*. Subsequently, the sponges together with the mussels were cultivated in aquaria in artificial sea water (Tropic Marine, Tropic Marine Centre Ltd, Rickmansworth; UK) in Mainz (Germany) at 17°C [37]. Those animals which had been used for the experiments were transferred into artificial sea water (pH 8.2; 20 mM Tris/HCl, containing 7 mM Na_2_SO_4_, 0.2 mM NaHCO_3_, 10 mM KCl, 540 mM NaCl, 50 mM MgCl_2_, and 10 mM CaCl_2_), as described [38]. Routinely, 10 specimens were placed on shells into 1 L aquaria. The sponges are fed as follows: every second d 0.2 mL/aquarium of ‘Coraliquid’, a fluid plankton-based nutrition (Sera; Heinsberg-Dohse, Bonn; Germany), 0.1 mL/aquarium of ‘Mainvit-plus’, containing strontium, molybdate, lithium, rubidium and iodine (Sera, Heinsberg; Germany) were added together with 0.05 g of deep-frozen *Artemia salina* (Hundt, Wuppertal; Germany). Where indicated, the specimens were cultured in aquaria at a lower concentration of CaCl_2_, using only 1 mM CaCl_2_ instead of the normal 10 mM concentration found in the oceans [38]. Routinely the incubation period was 5 d.

**Permissions:** No specific permits were required for the described field studies (locations/activities). The location of the collection site is not privately-owned or protected in any way. The field studies did not involve endangered or protected species.

### Spicules

The spicules were prepared from complete specimens and isolated as follows. Freshly collected specimens were soaked in 5% [v/v] NaOCl at room temperature as described [39]–[41]. Limited purification was achieved after a submersion period of 1 h. Then the spicules were collected on 0.2-µm pore-size nitrocellulose filters (Millipore, Schwalbach; Germany) and washed with distilled water. Those spicules were termed “sheath-spicules”. A complete removal of the organic surface layer was achieved by treating the spicules for 6 hrs with 5% NaOCl; “purified-spicules”. In order to ensure that all organic matter had been removed from the surface of the spicules, the samples were inspected under a light digital microscope (VHX-600 Digital Microscope from KEYENCE [Neu-Isenburg, Germany]), equipped with a VH-Z25 zoom lens.

### Scanning electron microscopy

Scanning electron microscopic (SEM) had been performed as described [42]. The samples were mounted onto aluminum stubs (SEM-Stubs G031Z; Plano, Wetzlar, Germany) which had been covered with adhesive carbon (carbon adhesive Leit-Tabs G3347). The SU 8000 microscope (Hitachi High-Technologies Europe, Krefeld, Germany) was employed at low voltage (<1 kV). The beam deceleration mode was used in order to improve the scannings [43].

### MTT sponge cell viability assay

The established MTT (3-(4,5-dimethylthiazol-2-yl)-2,5-diphenyltetrazolium bromide; Sigma, Taufkirchen; Germany) assay system, optimized for the determination of sponge cells, had been applied [44]. In brief, specimens after incubation in CaCl_2_-rich medium (10 mM CaCl_2_) or in CaCl_2_-depleted medium (1 mM CaCl_2_) were collected and immediately dissociated to single cells using Ca^2+^- and Mg^2+^-free sea water. From the suspensions, defined aliquots of 100 µL each (8×10^5^ cells/mL [adjusted with a hemocytometer]) were added to the wells and the cells were reacted with MTT (0.5 mg/mL). Finally, the optical densities were measured at 495 nm (595 nm as reference wavelength) in an ELISA Reader (model 450; Bio-Rad).

### Phage peptide display screening

Spicule-binding peptides were identified and then selected by using the Ph.D.-12 Phage Display Peptide Library Kit (New England Biolabs, Beverly, MA) following the described protocols [45], [46]. Equal amounts of two different types of commercially available library suspensions, 7mer and 12mer [PhD-7, and PhD-12] were mixed to obtain a diverse and randomized peptide library of >5×10^11^ pfu. The purified-spicule fraction, stored in TBS buffer (50 mM Tris-Cl, 150 mM NaCl; pH 7.6), were used for screening. The phage libraries were incubated with 1 mg spicules for 1 h in 1 mL of 0.1% Tween-20 in TBS (TBST) buffer. Subsequently, non-bound or loosely bound phages were removed by washing with TBS buffer during 10 cycles; the tightly bound phages were eluted with 1 mL of 0.2 M glycine-HCl buffer (pH 2.2) and neutralized with 1M Tris-HCl (pH 9.1). The phages eluted from the spicules were amplified in *Escherichia coli* cultures and collected by polyethylene glycol precipitation, as described in the instruction booklet (Ph.D.™ Phage Display Libraries; NEN). Amplified phage stocks were titrated and diluted to a concentration of 10^6^ pfu/µL. This selection procedure was sequentially repeated, but washing was done with 0.5% Tween-20 in TBS. After three rounds of screening, the eluted phages were plated, small cultures were inoculated by individual plaques, grown overnight, and the phage DNA was isolated for sequencing. Over 100 clones were picked and analyzed. With this approach, very likely, peptides are identified that they directly bind to ACC.

### Qualitative ELISA

The established ELISA [enzyme-linked immunosorbent assay] system had been applied to verify selective binding to spicule substrate [45]. In brief, stocks of selected phages (5×10^11^ pfu) were applied to purified-spicules in 100 µL of TBST and incubated for 37°C for 1.5 h. After incubation supernatant was removed, sediments were washed with TBST 6 times, blocked by 500 µL of 5% (w/v) skimmed milk in TBST for 1 h at 37°C. Then, 100 µL of a 1∶1000 dilution of rabbit anti-M13 antiserum in blocking buffer was added and continued to incubate for 1 h at 37°C. Thereafter, the solution was removed and the spicules were washed 3 times with 1 mL of TBST. 100 µL of 1∶2000 dilution of alkaline phosphatase labeled goat anti-rabbit antiserum (Sigma) in TBST was added, incubated at 37°C for 1 h. Finally, the immunocomplexes were detected with 200 µL of o-phenylenediamine dihydrochloride [0.4 mg mL^−1^] and the absorbance was read at 450 nm.

### Cloning of the two *S. raphanus* molecules

Two peptides had been identified by the phage display approach which comprises the sequence GDELSFDEGDVL, termed: Sycon-09, and TNMTMSNNGHSV, termed: Sycon-23]), respectively (see under “Results”). Based on the criteria outlined under “Results” Sycon-09 had been ascribed to an OSTF sequence and Sycon-23 to a carbonic anhydrase.

Searching the *S. raphanus* EST database (https://octavia.vk.medizin.uni-mainz.de/login.cgi), fragments of nt [nucleotide] EST sequences existed than have been used for completion of the respective cDNAs. The PCR [polymerase chain reaction] products were sub-cloned in the *pGEM-T* vector (Promega, Heidelberg; Germany) and, by that, the cDNAs were completed by primer walking [47].

The complete cDNA *SROSTFr* was 919 nts (excluding the poly(A) tail) long, comprised an 211 aa [amino acid] long ORF [open reading frame], encoding the putative protein OSTFr_SYCON.

In parallel, the complete carbonic anhydrase sequence was completed. The Sycon-23 peptide from the phage display screening was used as a start. Again using the *S. raphanus* EST database the complete cDNA was obtained by primer walking. The complete cDNA was 1,476 nts long (*SRCA*) comprising one ORF of 312 aa (CA_SYCON).

### Sequence analyses

Homology searches were conducted through servers at the European Bioinformatics Institute, Hinxton, United Kingdom and the National Center for Biotechnology Information (NCBI), Bethesda, MD. Multiple alignments were carried out with ClustalW version 1.6 [48]. Phylogenetic trees were constructed on the basis of aa sequence alignments applying the Neighbor-Joining method to distance matrices which were calculated using the Dayhoff PAM matrix model [49]. The degree of support for internal branches was further assessed by bootstrapping [50].

### Deposition of sequences

The following sequences from *Suberites domuncula* have been deposited (EMBL/GenBank); the cDNA for the osteoclast-stimulating factor (*SROSTF* gene) under the accession number HE610177, for carbonic anhydrase (*SRCA* gene) under HE610176, and for β1-tubulin (*SRTUBb* gene) under HE610178.

### Expression of carbonic anhydrase and OSTF and raising of antibodies

The two *S. raphanus* sequences, the carbonic anhydrase (cDNA *SRCA*; deduced protein CA_SYCON) and the OSTF (*SROSTFr*; OSTFr_SYCON), were expressed in *Escherichia coli* as described (Müller, et al. 2009a). In brief; a part of the carbonic anhydrase cDNA (*SRCA*), comprising the ORF from aa_17_ to aa_286_ [deduced size: 28,918 Da] and the complete ORF of the OSTF (*SROSTFr*) was expressed in the *E. coli* Gateway-Technology system in the *pDEST17* vector. After, in *pDEST17*, the clones were expressed in the *E. coli* (strain BL21-AI) (Invitrogen, Carlsbad,CA;USA), growing in LB medium, in the presence of 0.2% (w/v) L-arabinose for 12 h at 20°C. The bacterial pellets were then lysed with BugBuster according to the instructions of the manufacturer (Novagen/Merck KGaA, Darmstadt; Germany). After sonication the lysate was centrifuged. The insoluble fraction obtained was solubilized with the lysis buffer (50 mM KH_2_PO_4_, pH 8.0; 6 M urea, 300 mM KCl and 5 mM imidazole). Finally, the polyhistidine-tagged protein was purified by Ni-NTA Agarose affinity chromatography, according to the instruction booklet provided (Macherey-Nagel, Düren; Germany). The purified proteins were termed r-CA and r-OSTFr.

The polyclonal antibodies were raised as described [15] in female rabbits (White New Zealand). After the third boost the serum was applied for the experiments; the antibodies were termed PoAb-aCA and PoAb-aOSTFr. The titer of each antibody preparation was >1∶10 000. In controls, adsorbed sera were used which had been prepared as follows. PoAb-aCA or PoAb-aOSTFr (100 µL each) were incubated with 20 µg of the respective recombinant protein (r-CA and r-OSTFr). Those antibody preparations did not show a specific antigen-antibody complex on both Western blots and tissue slices (immunofluorescence analysis); data not shown.

### NaDodSO_4_-PAGE and Western blot analysis

Na-dodecyl sulphate polyacrylamide gel electrophoresis (NaDodSO4-PAGE) was performed, as described [51], [52]. The gels (10% polyacrylamide and 0.1% NaDodSO4) were stained with Coomassie brilliant blue.

After size-separation, the proteins were transferred to PVDF-Immobilon membranes according to Kyhse-Andersen [53] and described in details [52]. The antibody preparations were diluted to 1∶5,000 and used for the detection of the protein on blots (Western blotting). The immunocomplexes were visualized with anti-rabbit IgG (alkaline phosphatase conjugate; 1∶4000 dilution) together with the color develop system NBT/BCIP [nitro-blue tetrazolium chloride/5-bromo-4-chloro-3′-indolyphosphate p-toluidine salt] (Roth, Karlsruhe; Germany). In parallel, the antibodies were used for immunocytochemical analyses using tissue slices from *S. raphanus* that had been fixed in paraformaldehyde as described in details [54]. The immunocomplexes were visualized with Cy3-conjugated goat anti-rabbit immunoglobulin G (IgG). Parallel slices were stained with DAPI [4′,6-diamidino-2-phenylindole]. The slices were inspected with an Olympus AHBT3 microscope; the unstained slices were checked directly using Nomarsky interference contrast optics. The specimens were inspected under immunofluorescence light at an excitation light wave-length of 546 nm (Cy3-stained structures) or of 490 nm (DAPI).

### Histology

Tissue samples were sliced (5 µm thick) and stained with ASTRIN, as described [55]. Where indicated the tissue slices are inspected with Nomarski optics and fluorescent light [56].

### Quantitative real-time RT-PCR (qRT-PCR)

The expression of the two *Sycon* genes, *SROSTFr* and *SRCA*, was quantified by real-time RT-PCR (qRT-PCR) as described [57]. Following primer pairs were used: for the *Sycon* carbonic anhydrase (accession number HE610176): Fwd: 5′-TTGATGGCGGACAGAGGACTAC-3′ [nt_444_ to nt_465_] and Rev: 5′-AAGAGGACTCCAAACACGGAC-3′ [nt_567_ to nt_547_; size of the fragment, 124 bp], and the *Sycon* OSTF (HE610177): Fwd: 5′-AGCTGCAAAGCGATCAAACC-3′ [nt_406_ to nt_425_] and Rev: 5′-CGAAACGATGTCTTCTGCCC-3′ [nt_538_ to nt_519_; 133 bp]. As a reference, the *β-tubulin* gene from *S. raphanus* was used (HE610178): Fwd: 5′-GATAACGAGGCACTTTACGAC-3′ [nt685 to nt705], and Rev: 5′-GAGATCAGCATTGAGCTGAC-3′ [nt_828_ to nt_809_; size, 144 bp]. The sponge tubulin sequence shares (almost) 100% similarity for the deduced polypeptide to the corresponding β1-tubulins from *S. domuncula* (CAD79598.1), *Tribolium castaneum* (EFA06663.1) and *Xenopus laevis* (AAH54297.1). Mean Ct values and efficiencies were calculated by the iCycler software. The estimated PCR efficiencies vary within the range of 89%–105%. Expression levels of the respective transcripts - carbonic anhydrase or OSTF, were correlated with the one for ß-tubulin to assess the relative expression as follows [E_tubulin_^Ct tubulin^/E_carbonic anhydrase_^Ct carbonic anhydrase^; E_tubulin_^Ct tubulin^/E_OSTF_^Ct OSTF^, whereby “E” describes the PCR efficiency and “Ct” represents the threshold cycle [58].

### Statistical analysis

The results were statistically evaluated using the paired Student's t-test [59].

## Results

### The animals and their spicules

**Figure 1:**
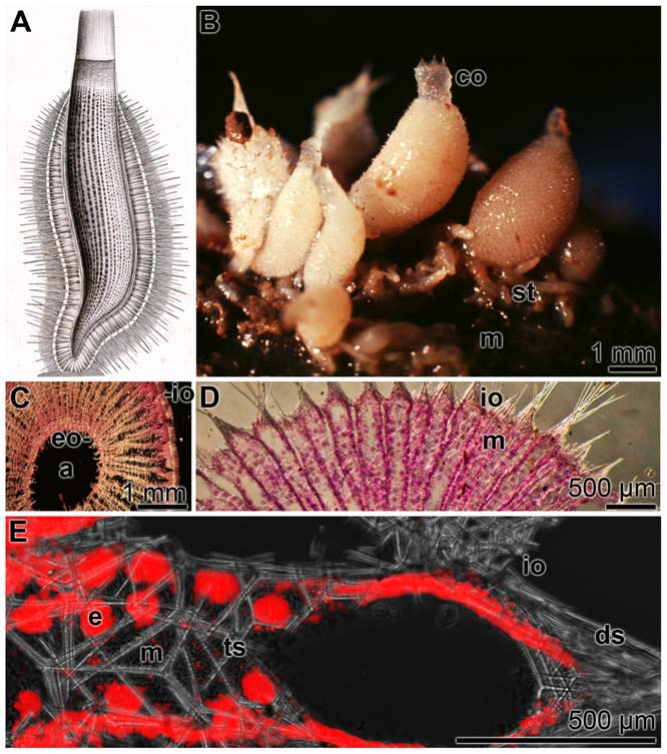
The calcareous sponge *Sycon raphanus* (Schmidt, 1862). (**A**) The species *S. raphanus* had been grouped by Haeckel [28] to the taxon *Sycandra*. Here a scheme of the morphology and the skeletal structure of *Sycandra hystrix* is given [28]. (**B**) *S. raphanus* specimens, growing on the mussel (m) *Mytilus galloprovincialis*. The oscule of the specimens is surrounded by a pronounced corona (co), formed of spicules. On the basis of the specimens stolons/buds (st) are seen. They develop after release from the parent sponge asexually to a descendent. (**C** and **D**) Cross section through *S. raphanus* specimens, displaying the external and internal surface layer. In the center the atrium (a) is shown into which the water canals flow in. Radial aquiferous canals traverse the body that originate at the surface of the animal, via the inhalant openings (io), and end at the internal surface via exhalant pores (eo). Between the canals the mesenchyme (m) compartments is radially arranged. The slices were stained with ASTRIN. (**E**) Non-stained section through the outer part of the sponge showing the location of the two major types of spicules; (i) the diactines spicules (ds), protruding from the distal cones of the outer surface of the specimens, and (ii) the triactines (ts) that are localized within the mesohyl. The mesohyl compartment is filled with eggs/embryos (e).

The calcareous sponge *S. raphanus* is morphologically grouped to the “syconoid sponges”, having a tubular body plan (Fig. 1A). The 5 to 10 mm long specimens of *S. raphanus* comprise a pronounced corona, formed of spicules that surround the oscule (Fig. 1B). Interspersed between the cortex and the outer cell layer of the sponge, of the specimens are the inhalant water canals that allow the water to be directed through the radial tube into the exhalant canals that flow into the atrium (Fig. 1C and D). From there, the water is pressed through the oscule into the external water environment. At the basis of the specimens, which are fixed to the substratum, stolons/buds are frequently formed that finally detach from the parent body after reaching sizes of around 2 mm; Fig. 1E.

**Figure 2:**
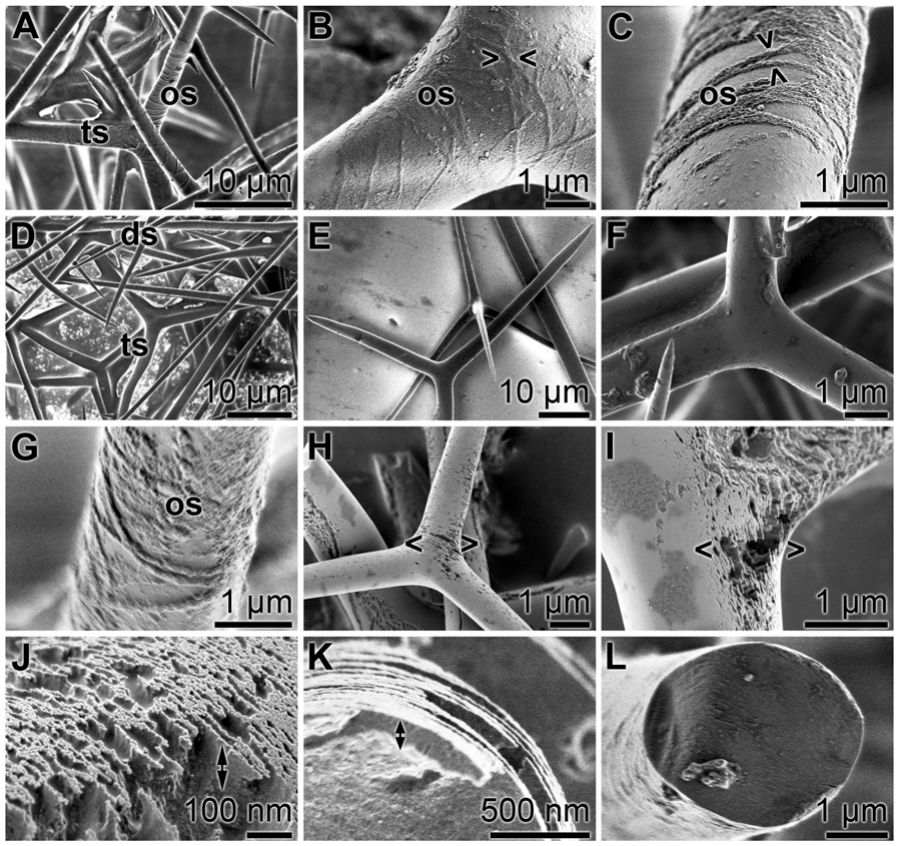
Surface architecture of spicules from specimens grown in normal ambient CaCl_2_ concentrations (10 mM) and cultivated in a CaCl_2_-depleted environment. The isolation of the spicules has been performed after a short exposure (1 h; sheath-spicules) or after an extended exposure (5 h; purified-spicules) to NaOCl. (**A**) Sheath-spicules from specimens grown in sea water, supplemented with 10 mM CaCl_2_. The surfaces of the abundantly occurring triactines (ts) are covered by organic sheaths (os). (**B** and **C**) At a higher magnification those layers, organic sheaths (os), can be resolved as circular netlike ropes whereby the individual fibrils apparently do not fuse to each other; they are tightly attached to the calcite surfaces (><). (**D** to **F**) Purified-spicules, diactines (ds) and triactines (ts), devoid of any visible sheath show a smooth surface. (**G**) Sheath-spicule from a specimen kept for 5 d in CaCl_2_-depleted aqueous environment, showing likewise an organic sheath. (**H** and **I**) Purified-spicules from similarly cultivated animals; the rough surface architecture is obvious (<>). (**J**) At higher magnification the rough surface architecture can be resolved as palisade bricks, sticking out about 100 nm radially from the spicules (double-headed arrow). (**K**) The fissuring of the spicules with an almost identical depth (double-headed arrow) suggests that the calcitic material of the spicules is not homogenous with respect to their density or content in organic material. (**L**) In comparison, the smooth surface of a purified-spicule, from an animal kept at 10 mM CaCl_2_, is shown.

Two types of spicules exist in *S. raphanus* [60]: the approximately 300 µm long diactines, forming short tufts that protrude from the distal cone of the outer surface of the specimens, and the about 300 µm large triactines and tetractines that are localized within the mesohyl (Fig. 1E). Considering the fact that the calcareous spicules are covered in the intact animals with a membranous, organic sheath [23], [24] a step-wise isolation procedure with NaOCl was applied. Firstly, “sheath-spicules” were prepared that retained their organic sheath to a substantial extent through a short (1 h) exposure treatment to NaOCl; secondly, “purified-spicules” were obtained that are devoid of any visible organic sheath by an extensive 5 h treatment with NaOCl. Those samples contain primarily triactines and to a lesser extent also tetractines, while only a small proportion of diactines exists. The surfaces of the sheath-spicules are covered and decorated with an organic layer (Fig. 2A) of a distinct fibrillar morphology (Fig. 2B and C). At a higher magnification those fibrils appear as circular netlike ropes that apparently do not fuse (Fig. 2C). The diameters of them range around 50 nm. Under the preparation conditions used, the fibrils intimately interact with the surface of the calcareous spicules. If the NaOCl-treatment of the spicules was extended, the spicules lose their organic sheath (Fig. 2D–F) and the surfaces become smooth; purified-spicules.

### Effect of lowering of the external CaCl_2_ concentration on spicule morphology

Specimens are kept in artificial sea water, which is depleted in CaCl_2_ (1 mM), for 5 d. Subsequently, the both sheath-spicules and purified-spicules are isolated and inspected by SEM. Like in animals kept at normal CaCl_2_ concentrations also, the specimens grown in CaCl_2_-depleted medium contain sheath-spicules (1 h in NaOCl) displaying a distinct organic sheath (Fig. 2G). However, if the treatment of the spicules with NaOCl is extended (5 h), the purified-spicules lose their smooth surface and become rough (Fig. 2H–K). Often a transition of the smooth to rough surface is seen especially at the centers of the spicules where the rays jointly originate (Fig. 2H and I). At higher magnifications the surface irregularities appear as palisade bricks, sticking out about 100 nm radially from the spicules (Fig. 2J). It is striking that the napped surfaces comprise protruding bricks that infiltrate into the spicules to an almost the same depth (Fig. 2K). This observation suggests that the calcitic scaffold of the spicules is not composed of a homogenous material, but is made of calcite of different density or organic/inorganic composition. In contrast to those purified-spicules from animals kept in a CaCl_2_-depleted milieu, the surfaces of the spicules from animals cultured at normal CaCl_2_ concentrations are smooth (Fig. 2L).

### Viability of the specimens kept in CaCl_2_-depleted aqueous environment

In order to assess the effect of lowering the CaCl_2_ concentration in the culture medium on the viability of the specimens, the MTT assay was applied, as outlined under “Material and Methods”. Animals were cultivated for 5 d either in the presence of the normal 10 mM CaCl_2_ in the medium, or in the CaCl_2_-depleted aqueous environment (1 mM CaCl_2_). Then their cells were obtained by dissociation in Ca^2+^- and Mg^2+^-free sea water. Subsequently the degree of viability was determined on the basis of the amount of formazan formation. The cell viability in the controls (10 mM CaCl_2_) was set to 100%. It was found that the viability of the control cells/animals was 100±15% (n = 10), compared to 96±17% in the CaCl_2_-depleted cells/animals, indicating that the difference between these two series is insignificant (P>0.5).

### Peptides bound to purified-spicules, identified by phage display

Over 100 five phage sequences had been obtained after sequencing. Among those two peptide sequences exist that had been most abundant: 15-times (GDELSFDEGDVL [termed: Sycon-09]) and 7-times (TNMTMSNNGHSV [termed: Sycon-23]).

Searching with the Sycon-09-GDELSFDEGDVL peptide for the most highly related sequence in the databases (BLAST Assembled RefSeq Genomes [http://blast.ncbi.nlm.nih.gov/Blast.cgi]), a series of protein sequences had been identified with an expect value E of approximately 0.3 [61]; besides of several hypothetical proteins, myosin and ankyrin domain containing polypeptides also, the OSTF 1 from the Jerdon's jumping ant *Harpegnathos saltator* had been identified (accession number EFN88679). Considering the fact that we already previously demonstrated that in the *S. domuncula* system, an OSTF is present that has a functional role in siliceous spicule formation [62], we focused on this molecule. We completed this sequence now also for *S. raphanus*, using the *S. raphanus* 8,000 ESTs containing data collection, as outlined under “Material and Methods”.

Analyzing the Sycon-23-TNMTMSNNGHSV peptide for the most highly related sequence in the sequence databases, the highest related sequence was identified with the hypothetical protein BRAFLDRAFT_127510 (accession number XP_002601262) from *Branchiostoma floridae* having an E value of 2.4e^−29^. Taking this *B. floridae* complete sequence as a basis and repeating the BLAST protein data base search again, the glycosyl-phosphatidylinositol-linked carbonic anhydrase from *Carcinus maenas* (ABX71209) was identified with E = 5e^−71^. In turn, searching the sponge base containing over 40,000 sequences (https://octavia.vk.medizin.uni-mainz.de/login.cgi) from *S. domuncula*, 28 clusters of ESTs sequences were found that encode deduced peptide fragments of the carbonic anhydrases. Also this cDNA sequence was completed.

**Figure 3:**
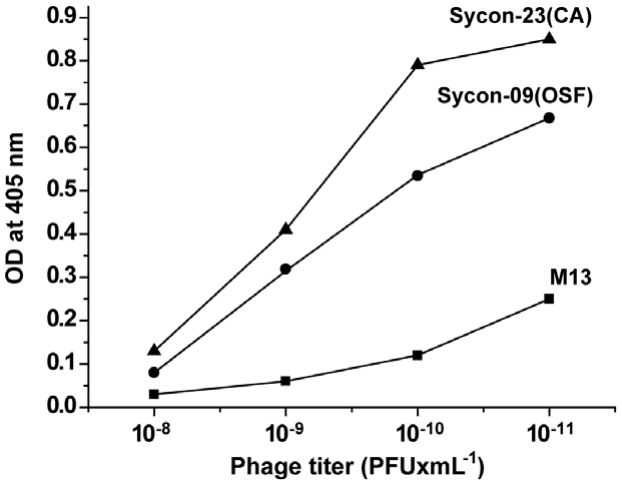
Results of the ELISA titration experiments with the phages Sycon-09 peptide, likely standing for the OSTF (Sycon-09(OSF)), and for the phage Sycon-23, indicative for the carbonic anhydrase (Sycon-23(CA)). The titer values (PFU/mL) obtained by using the antibodies against M13 revealed that the adsorption of Sycon-23(CA) and Sycon-09(OSTF) is much higher than the one read for the wild type phage M13.

The ELISA technique was applied to assess further the binding specificity of the phages. Dilutions had been performed with the clones Sycon-09 (relationship to OSTF sequence) as well as Sycon-23 (related to carbonic anhydrase) and reacted with antibodies against M13. The highest phage titer (PFU [plaque forming units]) was measured for the clone Sycon-23, followed by clone Sycon-09; the binding of the control phage M13 showed only at about two order of magnitudes lower a noticeable titer (Fig. 3). The background reaction of purified-spicules with anti-M13 antibodies was found at a titer of <10^−10^ (not shown).

### Cloning and expression of cDNA encoding the OSTF and the carbonic anhydrase

The two selected fragments obtained by phage display, indicative for the protein OSTF [Sycon-09(OSTF)] and for the carbonic anhydrase [Sycon-23(CA)], had been used as template to obtain the complete cDNAs, as described above.

#### OSTF

**Figure 4:**
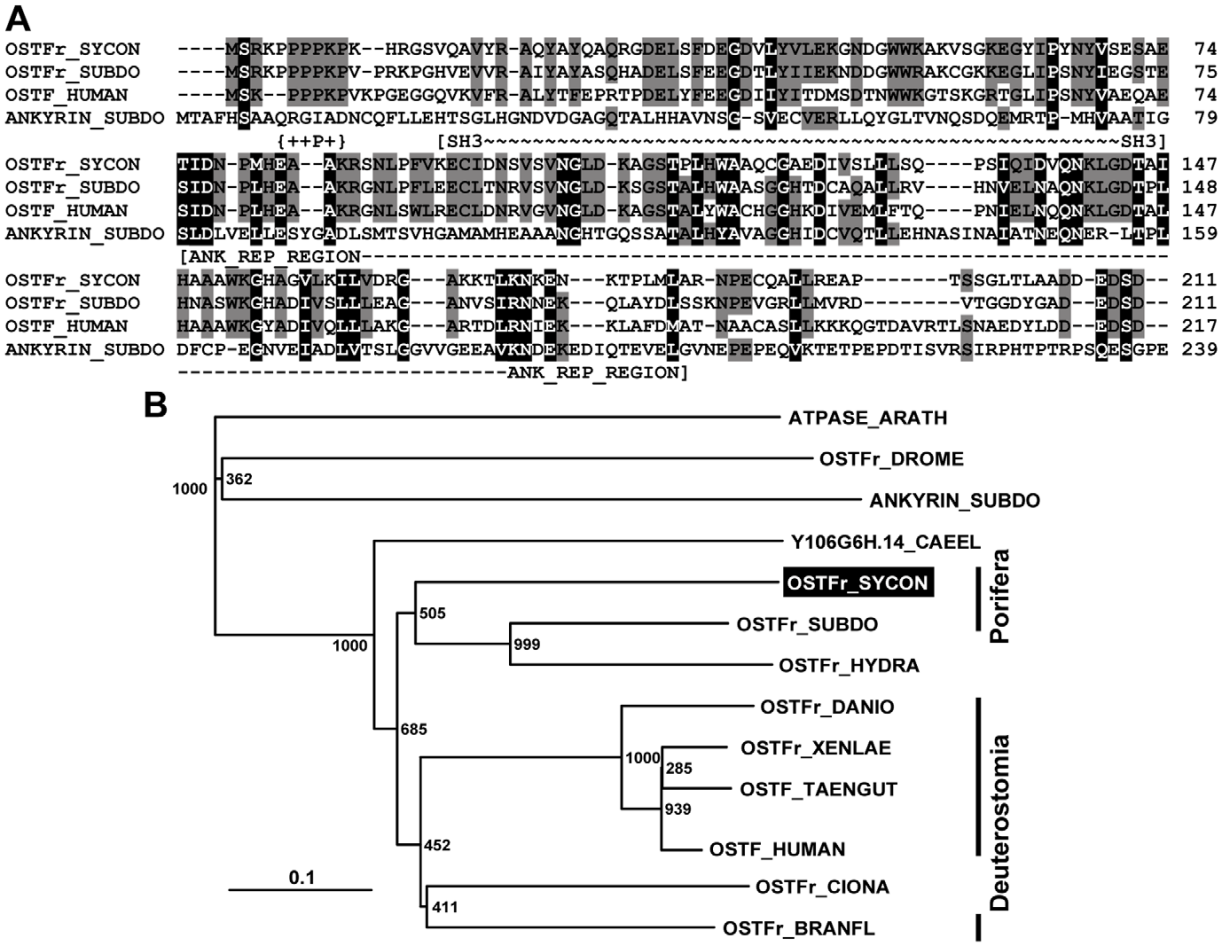
The *S. raphanus* putative OSTF (OSTFr_SYCON), deduced from the cDNA (*SROSTFr*). (**A**) The sponge OSTF was aligned with the related molecules from *S. domuncula* (OSTFr_SUBDO; CAJ44456.1) and human (OSTF_HOMO; EAW62571.1) as well as with the ankyrin sequence from *S. domuncula* (ANKYRIN_SUBDO ;CAH04634.1). The borders of the characteristic proline-rich region (+P+), the SH3 (∼SH3∼) as well as the domain ankyrin domain (-ANK_REP_REGION-) are marked. Residues conserved (identical or similar with respect to their physico-chemical properties) in all sequences are shown in white on black; those which share similarity between two sequences are in black on grey. (**B**) These four proteins were compared with the related polypeptide from *Hydra magnipapillata* (OSTFr_HYDRA; |XP_002165941.1), from *Branchiostoma floridae* (OSTFr_BRANFL; XP_002594542.1), from *Ciona intestinalis* (OSTFr_CIONA; XP_002126742.1), from the fish *Danio rerio* (OSTFr_DANIO gi|47086319|ref|NP_998022.1), from *Xenopus laevis* (OSTFr_XENLAE; NP_001080411.1), from the bird *Taeniopygia guttata* (OSTF_TAENGUT; XP_002190351.1), from the red deer *Cervus elaphus* (OSTFr_CERVUS; ABR68244.1), the distantly related sequences, hypothetical protein Y106G6H.14, from *Caenorhabditis elegans* (Y106G6H.14_CAEEL; NP_492738.1) and the ankyrin 2 from *Drosophila melanogaster* (OSTFr_DROME; NP_001189067.1). The tree was calculated and rooted with the plant sequence from *Arabidopsis thaliana* (ATPASE_ARATH; NP_178442.2), a proteasome non-ATPase regulatory subunit 10, as outgroup. Scale bar indicates an evolutionary distance of 0.1 aa substitutions per position in the sequence.

The full-length clone (*SROSTFr*) comprised one ORF spanning from nt_161–163_ to nt_791–793(without stop codon)_ and codes for a 211 aa long polypeptide, termed osteoclast-stimulating-related factor (OSTFr_SYCON); Fig. 4A. The deduced size [MW] of the polypeptide is 23,042 Da and the isoelectric point [pI] is 6.22. Domain search (http://prosite.expasy.org) revealed two segments within the OSTFr_SYCON polypeptide. At first, an N-terminal Src homology 3 (SH3) domain (spanning from aa_15_ to aa_74_; E = 4e^−19^) implies that the OSTFr_SYCON has putatively the ability to bind to proline-rich ligand(s), preferentially to ProxxPro motifs. Those peptide regions play a role in the regulation of enzymes by intramolecular interactions [63]. And second, an ankyrin repeat region profile within the region aa_75_ to aa_183_ (E = 8.26e^−36^), indicative that the protein comprises a protein∶protein interation domain [64]. Finally, at the N-terminus of the polypeptide a proline-rich region exists that is spanning the segment aa_15_ to aa_10_ (Fig. 4A). The sponge polypeptide shares 50%/66% identical/similar aa residues with the *S. domuncula* OSTF, 46%/67% identical/similar aa residues with the human OSTF and finally 11%/22% identical/similar aa residues with the *S. domuncula* ankyrin.

A rooted phylogenetic tree was constructed after alignment of the sponge and the human OSTF sequences, including also the additionally known related metazoan sequences (Fig. 4B). The tree shows that the two poriferan sequences form the basis for other metazoan polypeptides, including the OSTFs from *Hydra magnipapillata* and the invertebrate deuterostome, the cephalochordate *Branchiostoma floridae*, as well as the vertebrate deuterostomes (fish, bird, amphibian and mammal). This cluster does not include the protostomian sequence from *Drosophila melanogaster*, an ankyrin 2-related polypeptide. The latter sequence forms a separate branch with the sponge (*S. domuncula*) ankyrin sequence, reflecting that the two other segments within the *S. raphanus* OSTF polypeptide (the Src homology 3 (SH3) domain and the proline-rich part) essential determine the characteristics of the OSTFs.

#### Carbonic anhydrase

**Figure 5:**
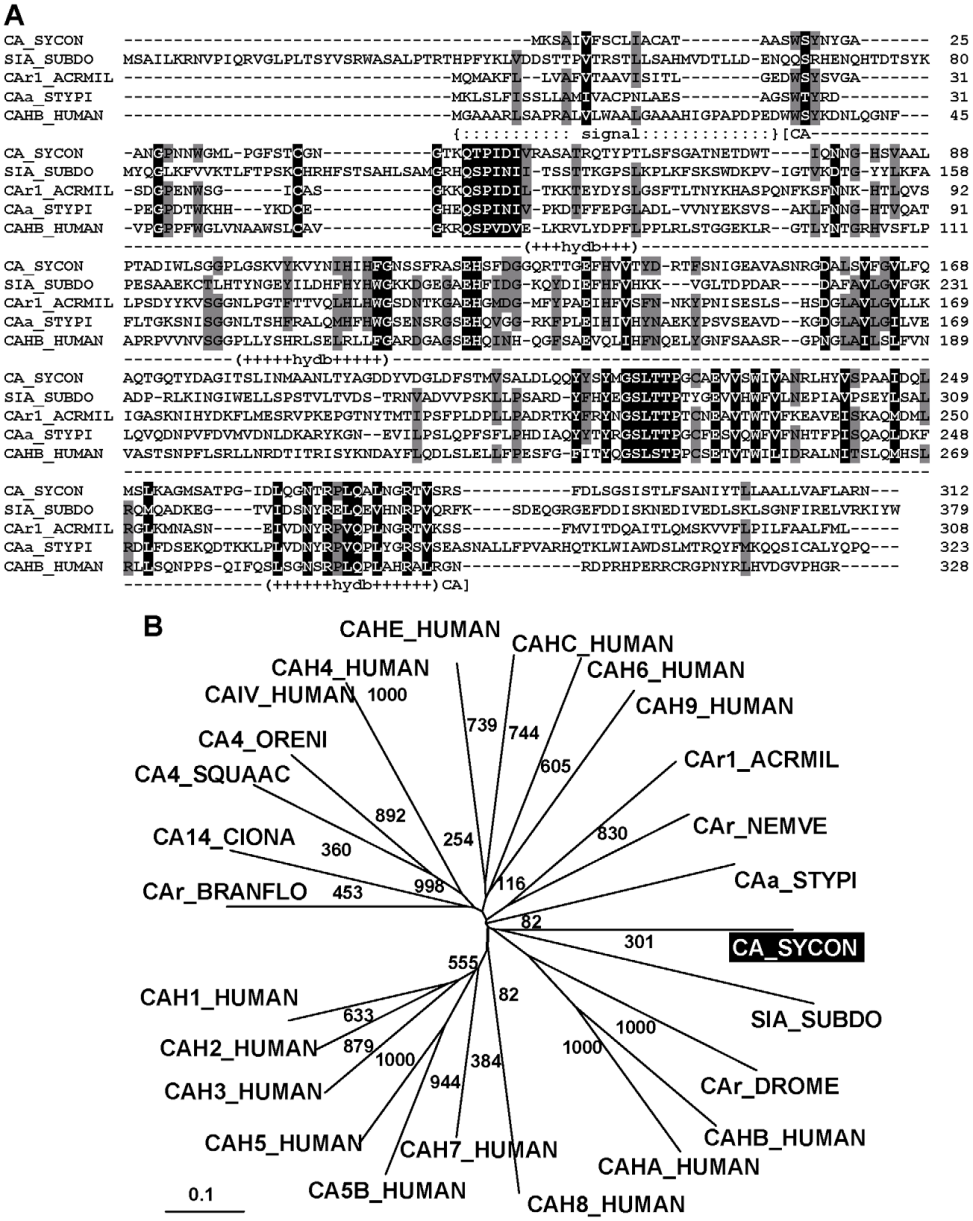
The *S. raphanus* putative carbonic anhydrase. (**A**) The sponge putative carbonic anhydrase (CA_SYCON) is aligned with the highly related sequences from the demosponge *S. domuncula*, the silicase (SIA_SUBDO; DD298191), and the carbonic anhydrases from the scleractinian *Acropora millepora* (CAr1_ACRMIL; ACJ64662.1), and the stony coral *Stylophora pistillata* (CAa_STYPI; ACA53457.1, EU159467.1), as well as with the human carbonic anhydrase 2 (CA II) (CAHB_HUMAN; O75493). The indicative sites/regions within the *Sycon* polypeptide are marked, the carbonic anhydrase alpha (vertebrate-like) group stretch (−CA−), including the His residues, functioning as Zn-binding sites, the hydrophobic parts (+hydb+), as well as the signal peptide (:signal:). Residues conserved (identical or similar) in all sequences are shown in white on black; those which share similarity to at least four residues are in black on grey. (**B**) Radial phylogenetic tree, including the mentioned sequences, together with human carbonic anhydrases of the following isoforms: I (CA-I) (CAH1_HUMAN; P00915); II (CA-II) (CAH2_HUMAN; P00918); III (CA-III) (CAH3_HUMAN; P07451); IV (CAIV_HUMAN; AAA35625.1); IV (CA-IV) (CAH4_HUMAN; P22748); VA (CAH5_HUMAN; P35218); VB (CA5B_HUMAN; CA5B_HUMAN); VI (CA-VI) (CAH6_HUMAN; P23280); VII (CA-VII) (CAH7_HUMAN; P43166); VIII (CA-VIII) (CAH8_HUMAN; P35219); IX (CA-IX) (CAH9_HUMAN; Q16790); 10 (CA-RP X) (CAHA_HUMAN; Q9NS85); XII (CA-XII) (CAHC_HUMAN; O43570); XIV (CA-XIV) (CAHE_HUMAN; Q9ULX7). In addition, the coral sequence from *Acropora millepora* (CAr2_ACRMIL; ACJ64663.1), as well as the ones from the sea anemone *Nematostella vectensis* (CAr_NEMVE; XP_001627923.1), the tunicate *Ciona intestinalis* (CA14_CIONA; XP_002123314.1); the lancelet *Branchiostoma floridae* (CAr_BRANFLO; XP_002601262.1), the shark *Squalus acanthias* (CA4_SQUAAC; AAZ03744.1); the fish *Oreochromis niloticus* (CA4_ORENI; XP_003456174.1), together with the insect enzyme from *D. melanogaster* (CAr_DROME; NP_572407.3) are included.

The complete 1,476 nts cDNA (*SRCA*) encodes, within its ORF (from nt_68–70_ to nt_1001–1003_), the 312 aa putative carbonic anhydrase (CA_SYCON), having a M_r_ of 33,251 and an Ip of 5.81. The prominent domain of the polypeptide (aa_17_ to aa_283_; E = 2e^−68^) is the carbonic anhydrase alpha (vertebrate-like) group stretch. This segment comprises the Zn-binding sites that are involved in the reversible hydration of CO_2_ enzymatic reaction (Fig. 5A). Those Zn-binding sites are complexed to the enzyme via their His residues [65]. A distinct signal peptide cleavage site exists between aa_16_ and aa_17_ (http://www.cbs.dtu.dk/cgi-bin/webface?jobid=signalp,4EE4B94B02FF3060&opt=none), indicative that the deduced enzyme is probably secreted or membrane-bound. Moreover, the ability to be membrane-associated can be deduced also from the sequence analysis, according to [66]; the three longer hydrophobic stretches predicted are marked in Fig. 5A.

The *Sycon* sequence share high sequence identity/similarity to the alpha-carbonic anhydrases from the *Acropora millepora* with a score of 25%/44% [67] and to *Stylophora pistillata* 23%/38% [68], [69]; similarly high is the relationship to the human carbonic anhydrase 2 with 19%/36%. The *A. millepora* and the *S. pistillata* carbonic anhydrases, have been grouped to the alpha type carbonic anhydrase family, and have been implicated in CaCO_3_ deposition from sponges to vertebrates [67]. Noteworthy is also the similarity (15%/29%) to the demosponge *S. domuncula* silicase, a carbonic anhydrase-related enzyme, that has been shown to mediate biosilica hydrolysis [70].

Since the protein structure of the metazoan carbonic anhydrases are different from those of the non-metazoan enzymes, an unrooted phylogenetic analysis has been performed with related sequences from Metazoa and the *Sycon* hypothetical enzyme (Fig. 5B). After alignment, those metazoan carbonic anhydrases were arranged according to their relatedness. This arrangement reveals that the *S. raphanus* is related to carbonic anhydrase branches within those enzymes identified in other diploblastic animals and the mentioned human alpha-carbonic anhydrase 2. More distantly related are the other known human isoforms of the carbonic anhydrase as well as the enzymes from the triploblastic protostomians.

### Production of recombinant carbonic anhydrase and OSTF and antibodies against them

The recombinant proteins of carbonic anhydrase and also of OSTF had been produced in *E. coli*, as described under “Material and Methods”. After expression of the respective cDNAs (*SRCA*; *SROSTFr*), the proteins were purified by affinity chromatography and termed r-CA or r-OSTFr. The samples were analyzed by NaDodSO4-PAGE.

#### Recombinant carbonic anhydrase

**Figure 6:**
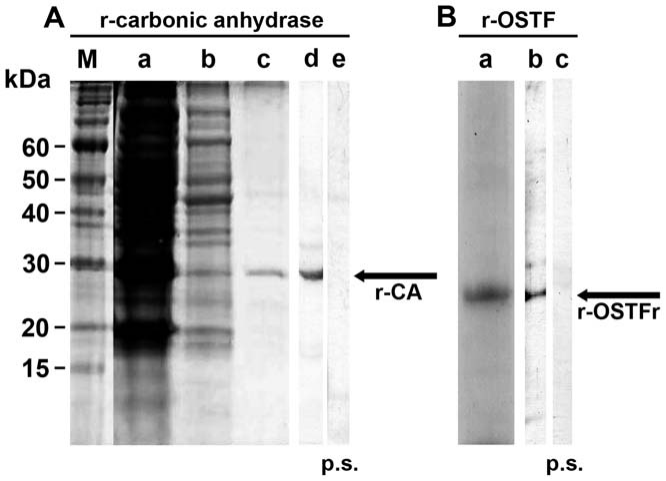
Production of recombinant *S. raphanus* carbonic anhydrase (r-CA) and OSTF (r-OSTF). (**A**) Preparation of the recombinant *S. raphanus* putative carbonic anhydrase (r-CA) and analysis by NaDodSO4-PAGE and Western blotting. NaDodSO4-PAGE: (**M**) Size markers. (**Lane a**) Proteins in the bacterial pellet, obtained from induced bacteria; (**lane b**) pattern after lysis with BugBuster; (**lane c**) affinity purified r-CA. Western blotting; (**lane d**) the antiserum raised against the r-CA (PoAb-aCA) recognizes the 29-kDa recombinant protein, while (**lane e**) a pre-immune serum (p.s.) did not react. (**B**) The recombinant OSTF protein (r-OSTF). (**Lane a**) NaDodSO4-PAGE analysis of the purified protein. Western blot analysis: (**lane b**) Reactivity of the antibodies raised against OSTF (PoAb-aOSTFr) to the 25 kDa r-OSTF; while (**lane b**) the pre-immune serum (p.s.) does not react.

The main segment of the *SRCA* cDNA was used for heterologous production of the oligohistidine-carbonic anhydrase fusion protein (r-CA) (Fig. 6A; lane a and b). The size of the purified protein was 28.5 kDa (lane c). The estimated value of this protein is 28,918 Da for the genuine sponge protein and, in addition, 930 Da more for the oligohistidine tag. Using this protein sample, polyclonal antibodies (PoAb-aCA) were prepared and have been found to be used to recognize the 29-kDa recombinant protein after separation of the recombinant protein by NaDodSO4-PAGE (lane d). This recombinant protein did not cross-react with a pre-immune serum (lane e).

#### Recombinant OSTF

Transfecting *E. coli* with the complete ORF of the *SROSTFr* cDNA, the recombinant OSTF (r-OSTFr) was purified in the same way. The protein shows a size of 24.5 kDa (Fig. 6B; lane a), matching with the calculated values (23.0 kDa [genuine protein] and 0.9 kDa [oligohistidine tag]). Again antibodies (PoAb-aOSTFr) were prepared; they recognized in the Western blot specifically the 24.5 kDa protein and (lane b); again the pre-immune serum did not show any reaction (lane c).

### Immunolocalization of the carbonic anhydrase and OSFT in sponge tissue

**Figure 7:**
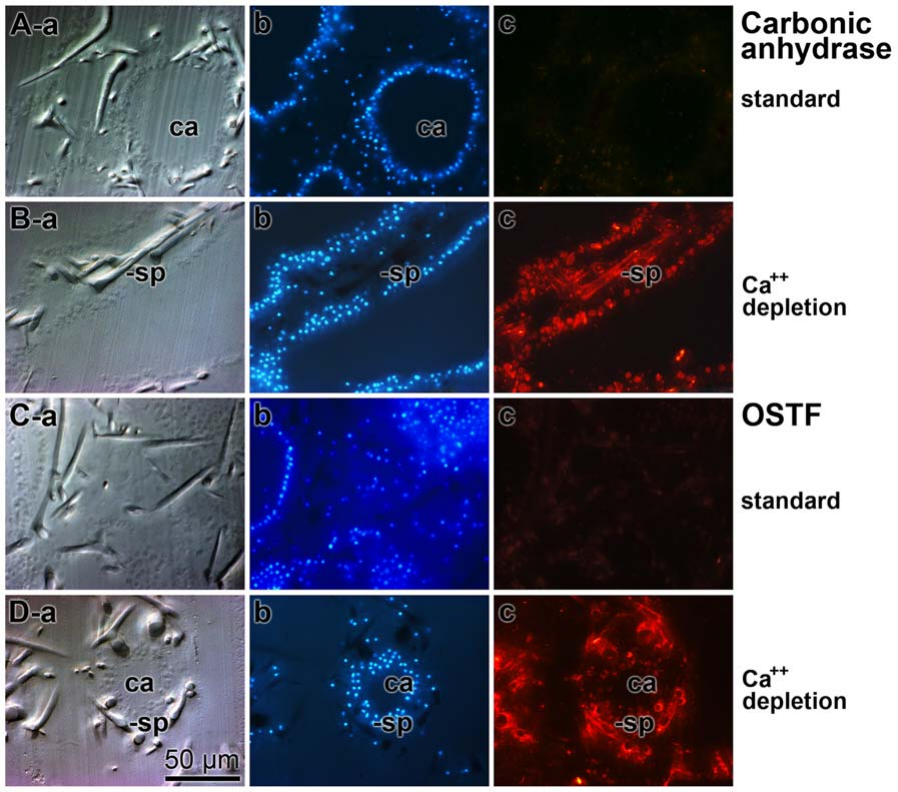
Localization and expression of carbonic anhydrase and OSTF in tissue from *S. raphanus*, kept under standard culture condition (containing 10 mM CaCl_2_) or under Ca^++^ depletion condition (1 mM CaCl_2_). Sections were performed through sponge tissue and stained with DAPI. In addition, the slices were reacted with one of the antibodies, either with PoAb-aCA (raised against recombinant carbonic anhydrase) or PoAb-aOSTFr (against OSTF). *First series:* Slices from specimens kept (**A**) under normal conditions were inspected either with Nomarsky interference contrast optics (**lane a**), or analyzed at 490 nm to localize DAPI staining (**lane b**) or at 546 nm to localize the carbonic anhydrase, based on the reaction of the PoAb-aCA antibodies. *Second series:* (**B**) Parallel series from a specimen, grown under Ca^++^ depletion condition; (**lane a**) analysis by Nomarsky optics, (**lane b**) for DAPI staining, or (**lane c**) for the localization of carbonic anhydrase. *Third series:* (**C**) Tissue slices through a specimen kept under normal conditions and inspected (**lane a**) with Nomarsky optics, (**lane b**) for DAPI staining, or (**lane c**) for OSTF using (PoAb-aOSTFr). *Fourth series:* (**D**) The parallel experiment performed with slices taken from a specimen, grown under Ca^++^ depletion; (**lane a**) Nomarsky optics, (**lane b**) for DAPI staining, or (**lane c**) for OSTF. In some images the canals (ca) and/or the spicules (sp) have been marked. The magnifications in all images are the same.

The two antibodies prepared (PoAb-aCA; and PoAb-aOSTFr) which specifically recognize the recombinant proteins carbonic anhydrase and OSTF on the Western blot were used to localize these two molecules in the sponge tissue, in dependence on the cultivation conditions, either standard condition (10 mM CaCl_2_), or Ca^++^-depletion (1 mM CaCl_2_). Reacting slices obtained from specimens, cultivated at standard condition, with PoAb-aCA gave no distinct staining pattern (Fig. 7A-c). The slice selected shows a cut through one canal running through the mesohyl, as seen by applying Nomarsky interference contrast optics and DAPI staining (Fig. 7A-a and -b). In contrast, if sections through a specimen were performed that had been cultivated under conditions of Ca^++^-depletion, the regions around the spicules as well as the spicules themselves are brightly stained with the antibodies (Fig. 7B-c). Nomarsky analysis or DAPI staining lighted up the spicules and also the cell rich regions within the mesohyl (Fig. 7B-a and -b). The cells at the borders to the spicules are termed sclerocytes; those cells probably reacted with the antibodies.

Using the antibody, prepared against the OSTF (PoAb-aOSTFr), almost the same pattern was seen. In tissue from animals living under standard condition (Fig. 7C), the reactions of the antibodies to structures within the slices were poor (Fig. 7C-c). Again, if tissue from animals living under Ca^++^-depletion condition, the regions around the spicules are intensively stained by PoAb-aOSTFr (Fig. 7D-c). In those regions where both the spicules and canals exist, the immunohistological analyses have been performed (Fig. 7C; Fig. 7D).

In parallel, controls had been performed with both adsorbed and with pre-immune serum of carbonic anhydrase and OSTF; in all assays no signals could be obtained in histological slices (data not shown).

### Decrease in steady-state level of mRNA expression during Ca^++^-depletion

The steady-state levels of expression of the two genes, the putative *carbonic anhydrase* (*SRCA*) and the *OSTF* (*SROSTFr*), have been determined in specimens, cultivated either under standard condition (10 mM CaCl_2_) or under Ca^++^-depletion condition (1 mM CaCl_2_). The quantifications have been performed by qRT-PCR and using the expression of the house-keeping gene *β-tubulin* as reference (Table 1 and Table 2). The transcript levels of both the *carbonic anhydrase* (Table 1) and the *OSTF* (Table 2) are low in animals living under standard condition, with approximately 0.5×10^−5^ and 0.3×10^−5^, respectively. However, already after an incubation period for 1 d at low Ca^++^ concentrations, the expression level increases significantly to 2.4×10^−5^ [*SRCA*] or 1.4×10^−5^ [*SROSTFr*]. Increasing the incubation time under Ca^++^-depletion condition to 3 or even 7 d, this resulted in a 14.7-fold increasing of the steady-state level for *SRCA* and 30.2-fold increasing for the *SROSTFr* gene.

**Table 1:** Expression levels of the putative carbonic anhydrase (*SRCA*) gene in *S. raphanus*.

Carbonic anhydrase	Gene expression(mRNA*_carbonic anhydrase_*/mRNA*_tubulin_*) ± SD	
Incubation period(d)	Standard condition(10 mM CaCl_2_)	Ca^++^-depletion(1 mM CaCl_2_)
0	0.31±0.02×10^−5^	0.91±0.07×10^−5^
1	0.58±0.02×10^−5^	2.43±0.18×10^−5^
3	0.94±0.04×10^−5^	9.82±0.41×10^−5^
7	0.85±0.05×10^−5^	13.28±0.92×10^−5^

The specimens were cultivated under standard condition (10 mM CaCl_2_) or at Ca^++^-depletion condition (1 mM CaCl_2_). After incubation periods of 3 to 7 d the animals were collected, RNA prepared and the extent of gene expression was quantified by qRT-PCR, as described under “Material and Methods”. Each data point represents the mRNA level of the respective expressed gene normalized to the amount of *β-tubulin* transcripts, as means ± SD (n = 6).

**Table 2:** Expression levels of the OSTF (*SROSTFr*) in *S. raphanus* in dependence on the level of Ca^2+^ in the culture medium.

OSTF	Gene expression(mRNA*_OSTF_*/mRNA*_tubulin_*) ± SD	
Incubation period(d)	Standard condition(10 mM CaCl_2_)	Ca^++^-depletion(1 mM CaCl_2_)
0	0.18±0.02×10^−5^	0.29±0.01×10^−5^
1	0.24±0.01×10^−5^	1.43±0.09×10^−5^
3	0.40±0.03×10^−5^	2.87±0.21×10^−5^
7	0.31±0.03×10^−5^	9.32±0.42×10^−5^

## Discussion

In the present study we followed a strategy, based on phage display of peptides, displaying with high affinities toward *S. raphanus* spicules, in combination with the existing EST libraries, to identify two potential polypeptides that might be associated with the surface of those skeletal elements. For these studies, “purified-spicules” from *S. raphanus* had been used after treatment with NaOCl for 6 h. While the spicules prepared after a shorter exposure period (1 h) [sheath-spicules] contained still the organic sheath, the purified-spicules, after exhaustive treatment, had been freed of that organic cover. The organic sheath appeared as circular fibrils that surround the spicules in a spiral-shaped manner. At the present state of our knowledge those fibrils can be homologized with the mesohyl-derived collagenous bundles [23], [71]. Beneath of the organic sheath, the surface of the spicules is smooth. However, after the more extensive treatment of the spicules with NaOCl, the surface of the spicules became fissured and palisade bricks are visualized. Those bricks infiltrate into the surface layer of the spicules suggesting that NaOCl can hydrolyze/remove the organic material that exists within the spicules [30], [72].

### Formation of rough surfaces onto spicules, formed by animals at low CaCl_2_ concentrations

In order to approach the ACC metabolism in *S. raphanus* the animals were kept in sea water containing low CaCl_2_ concentrations (1 mM) for 5 d. During this period the spicules retained their organic sheath, but drastically changed the morphology of the surface. In contrast to non-treated animals, those animals living in CaCl_2_-depleted condition were composed of spicules comprising a fissured surface. The surface is studded with palisade bricks that are directed outward from their calcareous rods. Under the conditions used the bricks are infiltrated almost homogenously only 100 nm, suggesting that the surface “layer” has a different chemical and/or organic composition. From the detailed studies of Aizenberg, et al. [73], it is known that the calcareous sponge spicules grow stepwise allowing proteins to control calcite formation, by intercalation into the crystals. Detailed time-dependent studies are in progress to resolve the NaOCl-caused dissolution of the ACC in the spicules.

### *In vitro* evidence for the participation of the OSTF and the carbonic anhydrase in spicular ACC

Purified-spicules had been used for the screening of expressed phage peptides that bind to those skeletal elements. Two peptides had been selected which displayed the highest binding affinity and had been most abundant during the screening process. The peptide binding was confirmed also by ELISA approach. Using those peptides and searching both the data bases available online (http://blast.ncbi.nlm.nih.gov/Blast.cgi) and the sponge base [EST] database (https://octavia.vk.medizin.uni-mainz.de/login.cgi) the two molecules, the carbonic anhydrase and the OSTF, had been identified.

The *S. raphanus* carbonic anhydrase shares the highest sequence similarity with the same enzyme (alpha-carbonic anhydrase) from the scleractinian coral *Stylophora pistillata* [68], [69] and also relationships to the α-carbonic anhydrase from the sponge *Astrosclera willeyana* [74]. This enzyme has been attributed to calcite formation and/or pH homeostasis in those animals. In addition, to those functional roles, the carbonic anhydrase has been implicated in the dissolution process of coral tissue proceeding during histocompatibility reactions in corals [75]. The role of the carbonic anhydrase as a calcium-based mineral-dissolving enzyme is extensively studied for vertebrates during bone resorption/remodeling [76], [77]. In both reactions, the enzyme acts through the generation of hydrogen ions from carbon dioxide during the CO_2_ hydration reaction. More specific, carbonic anhydrase-deficient animals show the phenotype of osteopetrosis [78], while overexpression of carbonic anhydrase has been implicated in osteoporosis [79]. More recently, the carbonic anhydrase has been shown not only to regulate intracellular pH milieu but also downstream bone dissolution in osteoclasts [80]. Interestingly enough, the *Sycon* carbonic anhydrase has been, like the coral (alpha-carbonic anhydrase) [68] and the osteoclast enzyme [80], predicted as a (likely) secreted and membrane-bound enzyme. In any event, the carbonic anhydrase catalyzes the interconversion of carbon dioxide and water to bicarbonate/CaCl_2_ and the release of protons [81]. Lowering of the pH finally results in the dissolution of CaCl_2_ [82], [83].

The *Sycon* OSTF is highly related to the previously identified factor from *S. domuncula* [62] and also to the human osteoclast stimulating factor 1 [84]. Like the human protein, the *Sycon* polypeptide also comprises the characteristic proline-rich region, the SH3 domain and ankyrin repeat(s). Since a signal peptide is missing, the factor is – like the related human one – an intracellular protein. In vertebrates this factor is selectively produced by osteoclasts, where it is enhancing osteoclast activity as well as the differentiation of those cells [84]. Based on studies with the mouse homolog of the factor, termed SH3P2, it is proposed that the molecule interacts with the cytoplasmic proto-oncogene product, c-Cbl that exists primarily as a tyrosine-phosphorylated protein in adhering cells together with a Src-family kinase [85], [86]. Binding of the OSTF to Src should occur via the proline-rich segment. In addition, the sponge factor comprises an ankyrin domain facilitating those protein∶protein interaction processes. In turn, a triple protein is formed [OSTF(SH3P2):Cbl∶Src] [87], suggesting that the OSTF interferes with the Src- and/or the Cbl-mediated pathways [86]. Those metabolic reactions that proceed in osteoclasts are integrated in the integrin-mediated signaling processes, during which the phosphorylated Cbl might become associated with the cytoskeleton. This translocation results finally in an association with the p85 subunit of the PI-3 kinase [phosphatidylinositol 3 (PI-3)-kinase] with Cbl [88]. After that, cell migration and cell spreading [89] is activated and the characteristics of membrane-associated lipid rafts are changed [90].

It is remarkable that the OSTF identified in the calcareous sponge *Sycon* shares also high sequence similarity to the factor present in the siliceous sponge *S. domuncula* [62]. In the latter animal system, the OSTF has been implicated in spicule resorption together with carbonic anhydrase-related silica dissolving enzyme silicase.

### Demonstration by immunohistology that both the carbonic anhydrase and the OSTF are associated with spicules

After having identified the complete cDNA, encoding these two molecules, in *S. raphanus*, antibodies had been prepared against them. The antibody preparations, anti-carbonic anhydrase and anti-OSTF, had been used to elucidate the localization of these proteins in animals. Analyzing tissue slices obtained from animals, grown under standard CaCl_2_ (10 mM) condition, hardly any signals can be identified for both PoAb-aOSTFr and PoAb-aCA. However, after cultivation of the animals at low CaCl_2_ (1 mM) condition for 5 d resulted in a drastic change of the staining pattern. The cells surrounding the spicules, the sclerocytes, become brightly stained and caused also an intensive contrasting of the spicules towards the adjacent tissue. Since the *Sycon* carbonic anhydrase is likely to be a secreted enzyme, it is conceivable that the enzyme can also be detected extracellularly by immunohistological analysis. However, the OSTF is an intracellular signaling molecule and the signals seen in the extracellular space are attributed to the destruction of the cell membranes during cutting the tissue and fixation afterwards.

### Gene expression studies of the molecules

qPCR analysis has been applied to quantify the steady state transcript levels of the putative carbonic anhydrase gene *SRCA* and also the gene encoding the OSTF, *SROSTFr*. This series of experiment confirms the immunohistological data. If the animals were kept under standard CaCl_2_ condition the expression level is low; it increases strongly, by 14.7-fold for the *carbonic anhydrase* gene and 30.2-fold for the gene encoding the OSTF, during the 7 d incubation period under CaCl_2_ - depleted condition.

These two sets of experiments convincing demonstrate that the expression of the genes encoding for the two sponge protein is upregulated after transfer of the animals to low CaCl_2_ concentrations. From the carbonic anhydrase it is well established that the expression level changes in response to microorganism settlement, like in plants [91], or to nutrient conditions, like in corals [68], [69], or CO_2_ sensing in animals [92]. No experimental data exist to indicate that the underlying gene for the OSTF is inducible; however, since this protein is expressed only at the final stage of the osteoclast development it is likely that the *OSTF* gene is differentially expressed during differentiation from hematopoietic stem cells to mature osteoclasts [93].

### Conclusion

We conclude that the genes encoding the two molecules, carbonic anhydrase and OSTF, are upregulated in *S. raphanus* specimens that had been kept under condition of CaCl_2_ depletion. In those animals – according to the SEM analyses – the spicules undergo fragmentation starting from the surface of the calcareous rods. Therefore, we have to assume that these two proteins become functionally active during especially the anabolic dissolution reaction of the ACC. Interestingly enough, these two molecules are highly conserved and are found in those vertebrate cells that are involved in bone-formation, more specific in osteoclasts rather than in osteoblasts, the function during bone degradation as well remodeling. The nomenclature of the different sponge cells, the cell typing, has only been started recently, by using molecular markers [94]. Therefore, both cells involved in spicule formation, in spicule dissolution and in spicule remodeling, have been collectively termed sclerocytes. For reasons of simplicity we also remain with this general nomenclature “sclerocytes” but denote them, according to their functional activity, as spicule-forming (anabolic sclerocytes) or the silica-degrading/remodeling cells (catabolic sclerocytes). In *S. domuncula*, sclerocytes had been identified as differentiated cells that originate from stem cells after exposure to inorganic ion, e.g. silicate or ferric ions, a process that involves the expression of noggin [94].

**Figure 8:**
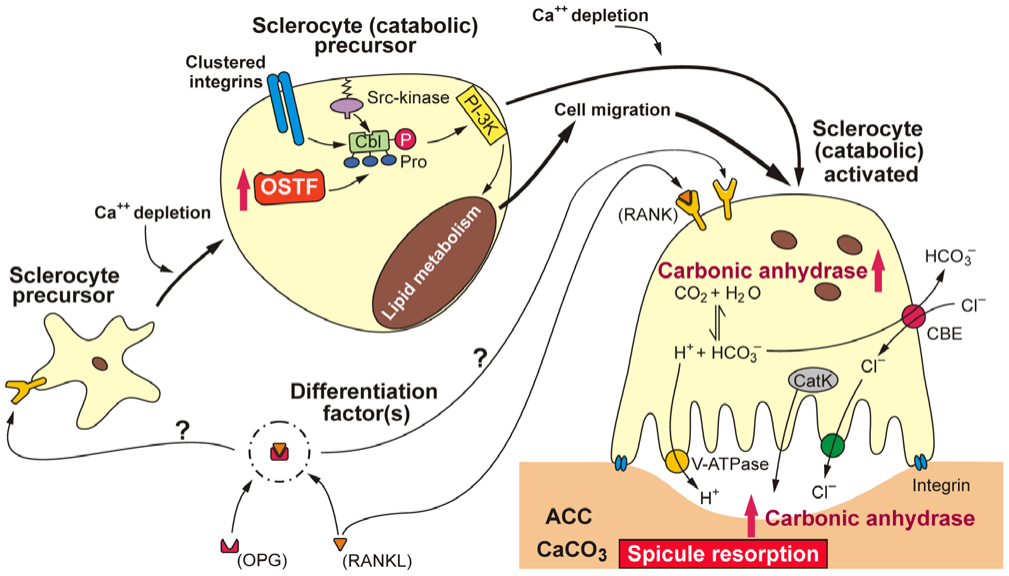
Model for the roles of the OSTF and the carbonic anhydrase in sclerocytes of the sponge *S. raphanus*. Based on the finding that the expression of the two molecules is upregulated during Ca^++^ depletion condition, it is proposed that these proteins are involved in the development of the precursor sclerocytes to the functionally active catabolic sclerocytes. OSTF forms with Cbl and Src a triple complex that stimulates the membrane-associated PI-3K and lipid metabolism. This metabolic chain is initiated by clustering integrins. These processes finally result in an increased mobility/migration of the catabolic sclerocytes and in an increased expression of the *carbonic anhydrase* gene. The latter enzyme generates protons that dissolve spicular CaCl_2_ (ACC), and causes spicule resorption. Presumably occurring pH shifts within the cells are counterbalanced by a vacuolar H^+^-transporting adenosine triphosphatase and the release of Cl^−^ via the chloride/bicarbonate exchanger (see: [58]). In analogy to the differentiation pathway of mammalian osteoclasts it is proposed that also in sponges the differentiation of sclerocytes is under control of, hitherto unknown, differentiation factors and their receptors acting similar like OPG, RANKL and RANK in mammals.

As a result of the presented data, primarily the immunofluorescence studies as well as the qPCR data, it is reasonable to propose that in response to Ca^++^ depletion the precursor sclerocyte cells differentiate to catabolic sclerocytes, a process during which the *OSTF* and also the *carbonic anhydrase* gene are upregulated. While the first factor stimulates the cell mobility, the activity of carbonic anhydrase (ultimately) increases around the catabolic sclerocytes and, in turn, causes an acid-driven dissolution of CaCl_2_ in the spicules In Fig. 8 the potential *in vivo* regulatory roles of OSTF and of carbonic anhydrase on the differentiation pathway of sclerocytes precursor cells to catabolic sclerocytes, mediating CaCl_2_ dissolution of spicules, it is sketched. It is a task for the future to identify the postulated differentiation factors controlling the differentiation of the sclerocytes cell lineage. In particular, we focus on the identification of cytokine(s) and their corresponding receptor(s), e.g. osteoprotegerin [OPG], receptor activator of NF-κB ligand [RANKL] and its receptor [RANK]. This cytokine/receptor triad crucially controls bone formation and bone remodeling in mammals [95].

In conclusion, with the present contribution, we open the window allowing an understanding of common features of biomineralization in calcareous sponges.
